# Seroprevalence and risk factors associated with brucellosis in humans and livestock in Nyagatare district of Rwanda

**DOI:** 10.3389/fpubh.2025.1665341

**Published:** 2025-09-26

**Authors:** Jean Bosco Ntivuguruzwa, Emmanuel Kabalisa, Happy Jean Bosco Asifiwe, Adeline Gapasi Uwamahoro, Immaculée Nyampinga, Agrippine Mukarurangwa, Nadine Rujeni, Jean Paul Habimana, Marie Louise Mukamuhirwa, Patience Karemera, Placidie Umukunzi, Beathe Iradukunda, Patrick Mazimpaka, Peter J. Hudson, Maurice Byukusenge, Joram J. Buza, Vivek Kapur, Adolphe A. Ndikubwimana, Isabelle Mukagatare, Martin Ntawubizi, Robab Katani

**Affiliations:** ^1^School of Veterinary Medicine, College of Agriculture, Animal Sciences and Veterinary Medicine, University of Rwanda, Nyagatare, Rwanda; ^2^National Reference Laboratory, Rwanda Biomedical Center, Kigali, Rwanda; ^3^School of Health Sciences, College of Medicine and Health Sciences, University of Rwanda, Kigali, Rwanda; ^4^School of Public Health, College of Medicine and Health Sciences, University of Rwanda, Kigali, Rwanda; ^5^Huck Institutes of Life Sciences, Pennsylvania State University, University Park, PA, United States; ^6^Department of Biology, Pennsylvania State University, University Park, PA, United States; ^7^Department of Veterinary and Biomedical Sciences, College of Agricultural Sciences, Pennsylvania State University, University Park, PA, United States; ^8^Nelson Mandela African Institution of Science and Technology, Arusha, Tanzania; ^9^Department of Animal Science, Pennsylvania State University, University Park, PA, United States

**Keywords:** brucellosis, sero-prevalence, risk factors, Rwanda, zoonotic, One Health

## Abstract

**Background:**

Brucellosis is a significant but under-reported bacterial zoonosis in Rwanda. Despite recognition as one of Rwanda's top six priority zoonotic diseases in 2019, comprehensive epidemiological data linking human and animal infections remain limited, particularly in high-risk pastoral communities. This study aimed to determine brucellosis seroprevalence and associated risk factors in humans and livestock in Nyagatare District, a major livestock-producing region of Rwanda.

**Methods:**

A cross-sectional study was conducted from March to October 2023 across three sectors (Karangazi, Rwempasha, and Rwimiyanga sectors) using stratified random sampling. Blood samples were collected from 886 humans and 930 livestock (637 cattle, 222 goats, 71 sheep) and screened via indirect Enzyme-Linked-Immunosorbent Assay. Risk factor data were collected through structured questionnaires. Multivariable logistic regression identified factors associated with seropositivity, with results expressed as odds ratios (OR) and 95% confidence intervals (CI).

**Results:**

The overall seroprevalence was 19.9% (176/886; 95% CI: 17.3–22.6) in humans and 10.9% (101/930; 95% CI: 9.0–13.0) in livestock. Among livestock, seroprevalence was highest in cattle (11.9%, 76/637; 95% CI: 9.4–14.5), followed by goats (11.3%, 25/222; 95% CI: 7.1–15.4), and sheep (1.4%, 1/71; 95% CI: 0.0–4.2). In humans, significant risk factors included male gender (OR = 2.66, 95% CI: 1.57–4.64, *p* < 0.001), age >55 years (OR = 7.39, 95% CI: 3.82–14.8, *p* < 0.001), and working as an animal health practitioner (OR = 2.90, 95% CI: 1.38–6.06, *p* = 0.005). In livestock, key risk factors included retention of aborted animals in herds (OR = 10.0, 95% CI: 2.27–49.2, *p* = 0.003), improper disposal of aborted fetuses (OR = 3.15, 95% CI: 1.18–7.99, *p* = 0.018), and shared water sources (OR = 2.49, 95% CI: 1.27–4.93, *p* = 0.008). Geographic analysis revealed higher seropositivity in the Rwimiyaga sector (OR = 3.06, 95% CI: 1.37–7.45, *p* = 0.009).

**Conclusions:**

This study reveals a high burden of brucellosis in both human and livestock populations in Nyagatare District, with particularly elevated risk among animal health workers and where livestock management practices are poor. Our findings suggest three targeted interventions: (1) Mandatory use of personal protective equipment for animal health workers, (2) Proper disposal of infectious animal materials, and (3) Sector-specific control strategies for high prevalence areas. These results provide critical evidence for developing One-Health interventions to control brucellosis in Rwanda and similar East Africa settings.

## 1 Introduction

Brucellosis represents one of the world's most widespread zoonotic diseases, causing significant human illness and substantial economic losses in livestock production (1, 2). The World Health Organization (WHO) has recognized brucellosis as a major neglected zoonotic disease that continues to pose significant public health challenges in endemic areas due to its impact on human health and livestock productivity, as well as difficulties in effective diagnosis, surveillance, and control measures (3).

The complexity of brucellosis is reflected in its diverse causative agents, with 12 recognized *Brucella* species classified according to host preference, pathogenicity, and phenotypic characteristics (4) Among these, six “core” species are particularly significant: *B. abortus* primarily affecting cattle, *B. melitensis* in goats and sheep, *B. suis* in pigs, *B. ovis* in sheep, *B. canis* in dogs, and *B. neotomae* in desert woodrats (5, 6). While these species show host preferences, cross-species transmission occurs frequently, with *B. melitensis* notably demonstrating broad host adaptability (7, 8).

Human infection primarily stems from three *Brucella* species: *B. abortus, B. melitensis*, and *B. suis* (7). Transmission to humans typically occurs through multiple pathways, including direct contact with infected animals, consumption of unpasteurized dairy products, and occupational exposure among veterinarians, livestock handlers, and abattoir workers. Airborne transmission also poses a significant risk (4). The World Health Organization estimates 500,000 new human cases annually (3) though this figure likely underestimates the true disease burden, particularly in malaria-endemic regions where symptoms may be misattributed (9).

Recent decades have seen successful control of *B. abortus* in many developed nations. However, the increasing prevalence of *B. melitensis* and *B. suis* in livestock has expanded both the geographical range and overall prevalence of human brucellosis (2, 10, 11). In developing countries like Rwanda, limited and inconsistent epidemiological data on *Brucella* species hampers effective disease control efforts. Comprehensive studies are essential to generate reliable incidence estimates, assess impact, understand transmission dynamics, identify reservoirs, and develop targeted control strategies (12).

To address these knowledge gaps, we conducted a cross-sectional study in Nyagatare District, a strategically important region bordering Tanzania and Uganda that contains 30% of Rwanda's cattle and goat populations (13). This district was specifically selected due to its high livestock density, pastoralist communities with frequent human-animal contact, and its transboundary location which increases risk of disease transmission across national borders. Our study had three primary objectives: 1) to estimate brucellosis seroprevalence in both human and livestock populations, 2) to identify risk factors associated with *Brucella* seropositivity, and 3) to assess awareness and practices related to brucellosis among high-risk occupational groups.

This research aims to provide comprehensive epidemiological data to inform evidence-based strategies for brucellosis prevention and control in Rwanda and similar settings, contributing to the broader goal of reducing the global burden of this zoonotic disease. By focusing on the human-animal interface in a high-risk region, our findings offer valuable insights for implementing effective One Health approaches to disease control in endemic areas.

## 2 Materials and methods

### 2.1 Study area and design

**Figure 1:**
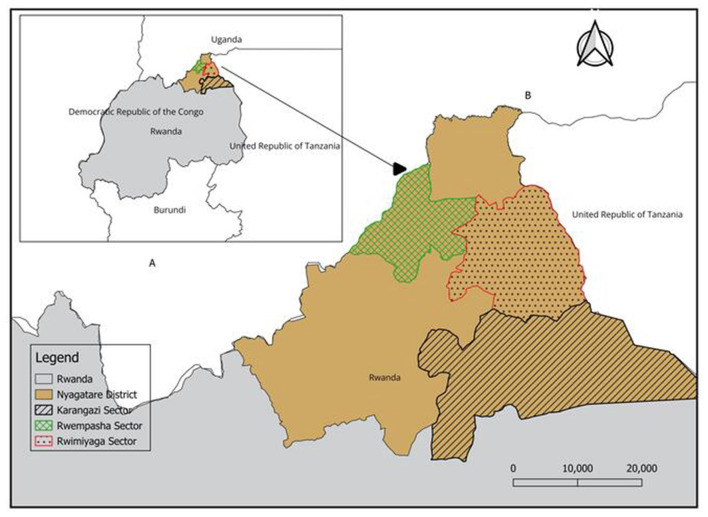
Map of Rwanda with neighboring countries **(A)**. Study area indicating Karangazi, Rwempasha, and Rwimiyaga sectors of Nyagatare district **(B)**. This map was created using a free online QGIS Desktop version 3.34.0. The spatial data (shapefiles) used are freely available from DIVA-GIS at https://gadm.org/download_country.html.

This research was conducted in Rwanda's Nyagatare District, Eastern Province, focusing on three administrative sectors: Karangazi and Rwimiyaga (adjacent to Akagera National Park and the Tanzanian border) and Rwempasha (bordering Uganda; Figure 1). Nyagatare District spans 1,920.11 km^2^ and hosts a population of 653,861 (14).The study area was selected based on its significance as Rwanda's primary livestock region, containing 30–40% of the country's cattle and goat populations, and its strategic location along international borders (13). The study region lies at approximately 1°8′0.00“S, 30°9′30.00”E, at an elevation of 1,513.5 meters above sea level. It experiences an average annual rainfall of 827 mm and temperatures ranging between 20 °C and 21 °C. The area's characteristic long dry season typically extends 3–5 months (May–June to August–September). The predominant semi-intensive livestock farming system operates within a landscape of gently sloping hills covered in grasslands (15).

### 2.2 Study design and sample size determination

This cross-sectional study was conducted from March to October 2023, using a stratified sampling approach to select individual human and animal subjects across three sectors. The human participants were selected from healthcare centers and community living in close proximity with livestock.

Before sampling, consultative meetings were held with the Nyagatare District Animal Resources Officer (DARO) and the District Director of Health (DDH) to gather information on livestock population distribution and to acquire a list of households engaged in livestock production. With the facilitation of DDH, the health centers located in the three selected sectors consented to participate in the study.

According to the information obtained from DARO and DDH, the population of cattle, goats, sheep, and humans in Nyagatare District was 431, 399; 150,392; 48,065; and 653, 861, respectively. Cattle represented 68.5% of the livestock population, followed by goats at 23.9%, and sheep at 7.6%. The animal subjects included in the study were cattle aged 1 year or older and small ruminants (sheep and goats) aged 5 months or older. The age of the livestock was determined using the dental formula (16). The sampling was conducted at the household level by selecting every fifth livestock-producing household. Human participants aged 18 years and above were sourced from the three selected Healthcare Centers and from the livestock community. At the healthcare centers, all human health care professionals and patients with symptoms of fever who voluntarily consented to participate in the study were enrolled in the study. In the livestock community, the animal handlers from each farm, milk handlers from each milk collection center, and meat handlers from abattoirs and butcheries who voluntarily consented to participate in study were enrolled.

The sample size was calculated using the Cochran's sample size formula for large populations (17),


(1) $$\begin{array}{l} Sample\;size\;\left(n\right)=\frac{Z^{2\;}P\left(1-P\right)}{d^{2}} \end{array}$$

where Z = 1.96 at 95% confidence level; P is the expected prevalence estimated to be 9.7% in humans and 10% in livestock based on previous studies (18, 19). The desired precision was estimated at 2% with 95% confidence interval. The calculated sample size was 842 in humans and 865 in livestock. The sample size was adjusted to account for an additional 5% margin of error, resulting in a total of 884 participants for humans and 924 for livestock. The livestock sample was then further stratified into three species—cattle, goats, and sheep—according to their proportions in the overall livestock population. The determined livestock sample size included 637 cattle, 222 goats, and 71 sheep.

#### 2.2.1 Design of the questionnaire and data collection

Demographic data, including participant identification and livestock details (e.g., owner's name, sample identification, age, sex, breed, and location) were recorded at the individual level for all selected subjects. A structured questionnaire (Supplementary material S1) was administrated to human participants through face-to-face interviews by trained research assistants fluent in both English and Kinyarwanda. These interviews were conducted in Kinyarwanda, the local language, to ensure clarity and comprehension. Prior to full deployment, the questionnaire was pilot-tested with 20 participants from a non-study area to ensure clarity, cultural appropriateness, and validity. Minor adjustments were made based on the pilot feedback.

The questionnaire for human participants assessed a wide range of potential risk factors including occupation, routine activities such as milking, herding, waste removal, and consumption of unpasteurized milk and undercooked meat. Knowledge and awareness about brucellosis transmission, symptoms, and prevention were also assessed.

For animal risk factors, the questionnaires covered various aspects, including herd size, composition, breeding methods, vaccination history, proximity to wildlife, grazing practices, access to veterinary services, and farmers' knowledge of the disease. To facilitate data collection and analysis, the questionnaires were handled using the Epicollect 5 software platform (20). This platform allows simultaneous collection of data and geographic coordinates (18).

#### 2.2.2 Blood sample collection and processing

Blood samples were collected from humans and livestock anonymously, with each sample identified only by a unique metadata code to maintain confidentiality. For human participants, approximately 4 mL of venous blood was aseptically drawn by a nurse from the arm using the venipuncture technique. In livestock, a veterinarian collected 8 mL of blood from the jugular vein in small ruminants and from the tail vein in cattle. All sample collectors received standardized training on proper collection, handling, and labeling procedures prior to the study commencement.

All samples were transported under a cold chain maintained at 4 °C to ensure their integrity. Upon arrival at the laboratory, the samples were centrifuged at room temperature for 10 min at 3,400 rpm. The serum was transferred into O-rings sealed tubes, properly labeled, and stored at −80 °C until further analyses. All analyses were subsequently conducted at the Rwanda National Reference Laboratory (NRL).

#### 2.2.3 Serological tests

##### 2.2.3.1 Indirect enzyme-linked immunosorbent assay (i-ELISA)

The initial identification of anti-*Brucella* antibodies was conducted using the Rose Bengal Plate Test (RBT; IDvet Innovative Diagnostics^®^, France), which was selected for its effectiveness in rapid, field-based screening. This test was used to screen farms as potential sites of interest. For more precise laboratory testing, the indirect Enzyme-Linked Immunosorbent Assay (i-ELISA) was employed due to its superior specificity and efficiency in confirming *Brucella* seroprevalence. Consequently, the reported findings are based exclusively on the i-ELISA results, which were utilized to assess risk factors and perform statistical analyses.

The detection of specific immunoglobulins against *Brucella* was carried out using a commercially available i-ELISA (IDvet Innovative Diagnostics^®^, Grabels, France) following the manufacturer's guidelines. Briefly, 10 μl of serum samples and ready-to-use positive and negative controls (PC and NC) provided in the kit were added to microtiter plates coated with the antigen. The assays were incubated at 25 °C for 45 min before the first wash was performed. Afterward, 100 μl of conjugate was added to each well and incubated for 30 min at 37 °C. Following a second wash to remove excess conjugate, the enzyme substrate was added, and the plates were incubated again at 37 °C for 15 min. The reaction was then stopped by adding 100 μl of stopping solution. The optical density (OD) of the samples was measured at 450 nm using a microplate photometer. We followed the kit's instructions to calculate S/P percentage values for controls and samples, using the formula: S/P = (OD_sample_ – OD_NC_/OD_PC_ – OD_NC_) × 100. To validate the test, the positive control's mean had to exceed 0.350 (OD_PC_ > 0.350), and the ratio of positive to negative control values had to be >3 (OD_PC_/OD_NC_ > 3). If these criteria were met, a sample's status was determined as follows: an S/P value of ≤ 120% indicated a negative result, while an S/P value of ≥ 120% indicated a positive result.

#### 2.2.4 Statistical analysis

The data from humans and livestock were analyzed separately using similar methodologies. Seroprevalence was calculated as the percentage of i-ELISA-positive samples for both human and livestock populations. Raw data, collected using Epicollect 5, were exported as comma-separated value (CSV) files. Data cleaning, structuring, and analysis were performed using R version 4.4.0 (21). For the analysis, cattle were classified into three age groups: young (1–2 years), young adults (3–4 years) and adults (≥5 years). Similarly, small ruminants were grouped into young (5–12 months), young adults (13–24 months), and adults (>24 months). We employed logistic regression to assess associations between potential risk factors and *Brucella* seropositivity. The analysis followed a two-step approach to evaluate the risk factors associated with Brucella seroprevalence: 1) Univariable analysis: potential risk factors were individually evaluated for their association with *Brucella* serological status using simple logistic regression. This step identified variables with significant associations (*p* < 0.05) for further consideration. 2) Multivariable logistic regression: a multivariable model was constructed to account for potential interactions and confounding among predictors. Initially, all identified potential risk factors with *p* < 0.2 in univariable analysis were used to fit the model using the Generalized Linear Model (GLM) function with a binomial distribution. Model selection was performed using a backward stepwise approach based on the Akaike Information Criterion (AIC) to determine the best-fitting model.

The final model's fit was evaluated using K-fold cross-validation implemented through the “boot” package in R. Predictive accuracy was quantified using the area under the Receiver Operating Characteristic (ROC) curve (AUC) calculated with the “pROC” package. For each risk factor, we calculated odds ratios (OR) and 95% confidence intervals (CI). Statistical significance was set at *p* < 0.05.

#### 2.2.5 Ethical considerations

The study protocol was approved by the Institutional Review Board of Pennsylvania State University (IRB-PSU, STUDY00018709) and the Institutional Animal Care and Use Committee (IACUC-PSU, PROTO202101993). The study protocol was reviewed and approved by the Rwandan National Ethics Committee (No.453/RNEC/2022). In addition, administrative approvals were obtained from the Rwanda Biomedical Center (RBC) and the Nyagatare District Office.

All research procedures adhered to the guidelines and regulations prescribed by these regulatory committees. For human participants, written informed consent was obtained from all adults (18 years and above) prior to survey administration and sample collection. Participants were informed about the study's purpose, procedures, potential risks and benefits, and their right to withdraw at any time without consequence.

For livestock sampling, we followed the guidelines outlined in the Animal Welfare Act and the Institute for Laboratory Animal Research Guide for the Care and Use of Laboratory Animals. Efforts were made to minimize animal discomfort during sample collection.

All personal identifiers were removed from the data to ensure participant confidentiality. Data were stored securely with access restricted to authorized research team members.

## 3 Results

### 3.1 Seroprevalence and potential risk factors for brucellosis in human participants

#### 3.1.1 Descriptive statistics and univariable regression analysis results for human participants

A total of 886 human participants were included in this study, comprising 665 males (75.1%) and 221 females (24.9%). Participants were distributed across three sectors: Rwempasha (*n* = 315, 35.6%), Rwimiyaga (*n* = 344, 38.8%), and Karangazi (*n* = 227, 25.6%). Screening via i-ELISA method revealed an overall brucellosis seroprevalence of 19.9% (95% CI: 17.3–22.6).

The occupational roles of participants were categorized as follows: livestock farmers (*n* = 442, 49.9%), healthcare practitioners (*n* = 36, 4.1%), veterinary professionals (*n* = 54, 6.1%), meat handlers (*n* = 153, 17.3%), milk handlers (*n* = 186, 20.1%), and individuals in other occupations, such as teachers (*n* = 15, 1.7%). Additionally, 195 individuals were patients visiting healthcare facilities, with 56.9% presenting with a fever of unknown origin (Supplementary material S1 Questionnaire).

#### 3.1.2 Univariable analysis of risk factors for human participants

Univariable logistic regression identified multiple demographic and behavioral factors significantly associated with brucellosis seropositivity (Supplementary Table S2). Males had higher odds compared to females (OR = 2.66, 95% CI: 1.57–4.64). Increasing age was strongly associated with infection: 36–45 years (OR = 2.88, 95% CI: 1.56–5.47), 46–55 years (OR = 4.89, 95% CI: 2.62–9.41), and >55 years (OR = 7.39, 95% CI: 3.82–14.8), compared to the 18–25 years reference group. Occupational exposure also influenced risk: animal health practitioners had greater odds compared to livestock farmers (OR = 2.90, 95% CI: 1.38–6.06). Behavioral practices such as herding livestock (OR ≈ 2.30, 95% CI: 1.60–3.30), assisting in parturition (OR ≈ 2.00, 95% CI: 1.30–3.00), handling aborted fetuses (OR ≈ 3.300, 95% CI: 2.20–4.90), and contact with livestock waste (OR ≈ 2.50, 95% CI: 1.80–3.60) were all strongly associated with higher seropositivity. In contrast, consumption of raw milk (OR ≈ 1.20, 95% CI: 0.80–1.70) or raw milk products did not show significant associations. These findings suggest that direct occupational and livestock-handling exposures were the primary drivers of risk.

#### 3.1.3 Multivariable regression analysis results for human participants

The results of the multivariable logistic regression analysis, presented in Table 1, identify several statistically significant risk factors for *Brucella* seropositivity. A backward stepwise elimination based on Akaike Information Criterion (AIC) minimization was used to ensure the best model fit. Variables identified as statistically significant risk factors include gender, age, and occupational activity. Gender emerged as a significant factor, with males showing higher odds of seropositivity compared to females (OR = 2.66, 95% CI: 1.57–4.64, *p* < 0.001). Age also played a crucial role, with the odds of seropositivity increasing significantly across age groups (*p* < 0.001). Compared to the youngest age group (18–25 years), individuals aged 36–45 years (OR = 2.88, 95% CI: 1.56–5.47), *p* < 0.001), 46–55 years (OR = 4.89, 95% CI: 2.62–9.41), and individuals over 55 years (OR = 7.39, 95% CI: 3.82–14.8) were at progressively higher risk relative to those aged 18–25 years. Occupation also played a significant role, with individuals in animal health care professions being at higher odds of seropositivity (OR = 2.90, 95% CI: 1.38–6.06, *p* = 0.005) compared to livestock farmers.

**Table 1:** Multiple regression analysis of the potential risk factors for human brucellosis.

**Characteristic**	**OR ^a^**	**95% CI ^b^**	***p*-value**
**Gender**			
Female (ref)	—	—	
**Male**	**2.66**	**1.57–4.64**	**< 0.001**
**Age**			
18–25 (ref)	—	—	
26–35	1.55	0.85–2.91	0.200
**36–45**	**2.88**	**1.56–5.47**	**< 0.001**
**46–55**	**4.89**	**2.62–9.41**	**< 0.001**
**Over 55**	**7.39**	**3.82–14.8**	**< 0.001**
**Occupation group**			
Livestock farmers (ref)	—	—	
**Animal health care practitioners**	**2.90**	**1.38–6.06**	**0.005**
Human health care practitioners	2.36	0.84**–**6.00	0.083
Meat handlers	0.73	0.35**–**1.48	0.400
Milk handlers	1.09	0.64**–**1.84	0.800
Others	3.99	0.79**–**15.60	0.062
**Contact with livestock waste**			
No (ref)	—	—	
Yes	1.60	0.97**–**2.64	0.065
**Contact with aborted fetuses**			
No (ref)	—	—	
Yes	1.57	0.94**–**2.64	0.085
**Milked livestock**			
No (ref)	—	—	
Yes	1.57	0.94**–**2.64	0.088

^a^OR, odds Ratio;

^b^CI, Confidence Interval.

Bolded values represent statistically significant risk factors.

The model's predictive performance was assessed using 10-fold cross-validation, yielding a cross-validation error (deviance) of 0.144, with a bias-corrected estimate of 0.144. The area under the ROC curve (AUC) was 0.76.

#### 3.1.4 Questionnaire results

Additional questionnaire responses indicated that 49.8% of participants reported consuming raw milk, while 14% reported eating raw meat, and 4% reported consuming raw animal blood (Supplementary Table S2). Awareness of brucellosis was low: only 28.7% had ever heard of the disease, and fewer than 5% knew it was zoonotic. The logistic regression analysis did not show a significant association between raw milk consumption and seropositivity (21.3% vs. 18.9%, *p* = 0.41; Supplementary Table S2).

### 3.2 Seroprevalence and potential risk factors of brucellosis in livestock

#### 3.2.1 Descriptive statistics and univariable regression analysis results for livestock

Overall seroprevalence of brucellosis in livestock was estimated at 10.9% (101/930; 95% CI: 8.96–12.98). Among the different livestock species, cattle exhibited the highest seroprevalence at 11.9% (76/637; 95% CI: 9.41–14.45), closely followed by goats at 11.3% (25/222; 95% CI: 7.10–15.40). Sheep showed a remarkably lower seroprevalence of 1.4% (1/71; 95% CI: 0.00–4.15).

Univariable logistic regression analysis identified several demographic and management-related factors significantly associated with Brucella seropositivity (Supplementary Table S3). Animals from the Rwimiyaga sector had higher odds of infection compared to Karangazi (OR ≈ 2.40, 95% CI: 1.50–3.90, *p* < 0.001), while those from Rwempasha had lower odds (OR ≈ 0.60, 95% CI: 0.30–1.00). Adult animals (≥5 years) showed higher odds of seropositivity (OR ≈ 5.00, 95% CI: 1.2–20.0) compared to young animals (1–2 years). Female animals were more frequently seropositive than males (OR ≈ 2.70, 95% CI: 1.00–7.50). Certain breeds, particularly crossbred cattle and goats, showed elevated odds compared to local or exotic breeds. Livestock health status was strongly associated: animals with a history of abortion or infertility had markedly higher odds of seropositivity (OR ≈ 3.30, 95% CI: 1.50–6.80).

Management practices also showed significant associations with seropositivity. Retaining aborted animals in herds was strongly linked with infection (OR ≈ 7.50, 95% CI: 3.50–15.90). Improper disposal of aborted materials increased risk: discarding in open pastures (OR ≈ 2.50, 95% CI: 1.40–4.30) compared to safe disposal methods. In contrast, burying aborted materials reduced odds (OR ≈ 0.40, 95% CI: 0.20–0.70). Breeding practices also mattered: use of bulls from outside the farm increased odds (OR ≈ 3.0, 95% CI: 1.10–8.20). Introducing new animals into herds (OR ≈ 1.60, 95% CI: 1.10–2.40) and acquiring replacements from markets or distant districts (OR ≈ 2.00, 95% CI: 1.10–3.60) also increased risk. Finally, lack of awareness of brucellosis among livestock keepers was associated with higher seropositivity (OR≈3.50, 95% CI: 1.20–10.30).

#### 3.2.2 Multivariable regression analysis results for potential risk factors of brucellosis in livestock

The multivariable logistic regression analysis revealed several significant factors associated with *Brucella* seropositivity in livestock (Table 2). These factors included geographic location (sectors), age, herd size, sharing watering points, handling and disposal of aborted fetal tissues, and the fate of animals that undergo abortions.

**Table 2:** Multiple logistic regression analysis of the potential risk factors of brucellosis in livestock.

**Predictors**	**OR**	**95% CI**	***p*-value**
**Sector**			
Karangazi (ref)	—	—	
Rwempasha	1.90	0.79–4.72	0.200
**Rwimiyaga**	**3.06**	**1.37–7.45**	**0.009**
**Herd size**			
**Cattle**	**1.02**	**1.01–1.04**	**0.008**
Sheep	0.97	0.94–1.00	0.100
Goats	1.01	1.00–1.03	0.200
**Farm fencing**			
No (ref)	—	—	
Yes	2.40	0.79–9.27	0.200
**Shared water sources**			
No (ref)	—	—	
**Yes**	**2.49**	**1.27–4.93**	**0.008**
**Feed animals with abort(s)**			
No (ref)	—	—	
**Yes**	**0.33**	**0.11–0.91**	**0.037**
**Throwing abort(s) in the pastures**			
No (ref)	—	—	
**Yes**	**3.15**	**1.18–7.99**	**0.018**
**Species**			
Cattle (ref)	—	—	
Goat	0.99	0.54–1.76	>0.900
**Sheep**	**0.12**	**0.01–0.60**	**0.042**
**Age**			
Young (ref)	—	—	
**Adult**	**9.26**	**1.82–17.00**	**0.033**
Young Adult	4.44	0.16–126.00	0.300
**Sex**			
Female (ref)	—	—	
Male	0.34	0.08–1.00	0.086
**Aborted animal fate**			
Not applicable (ref)	—	—	
**Kept in the herd**	**10.00**	**2.27–49.2**	**0.003**

Bolded values represent statistically significant risk factors.

Animals from the Rwimiyaga sector (OR = 3.06, 95% CI: 1.37–7.45, *p* = 0.009) were more likely to be associated with brucellosis seropositivity compared to those from the Karangazi and Rwempesha sectors. Adult animals (5 years and older) were more likely to be seropositive than young animals (OR = 9.26, 95% CI: 1.82–17.0, *p* = 0.033). Only in cattle the herd size was slightly associated with brucellosis (OR = 1.02, 95% CI: 1.01–1.04, *p* = 0.008), with larger herds being more associated with brucellosis seropositivity. Statistically significant risk factors associated with herd management and higher odds of *Brucella* seropositivity included keeping aborted animals in the herds (OR = 10.0, 95% CI: 2.27–49.20, *p* = 0.003), disposing of aborted fetal tissues in the open pastures (OR = 3.15, 95% CI: 1.18–7.99, *p* = 0.018), and sharing watering points (OR = 2.49, 95% CI: 1.27–4.93, *p* = 0.008) among livestock herds. Conversely, burying aborted fetal tissues (OR = 0.08, 95% CI: 0.02–0.26, *p* < 0.001) and feeding aborted fetal tissues to companion animals (OR = 0.33, 95% CI: 0.11–0.91, *p* = 0.037), were associated with lower odds of *Brucella* seropositivity. The model demonstrated strong predictive performance and stability, with AUC of 0.78 and a cross-validation deviance of 0.091 with a bias-corrected estimate of 0.090.

## 4 Discussion

Brucellosis remains an endemic disease affecting both livestock and humans in developing countries, posing significant public health and economic burdens (22, 23). Despite its classification as a neglected zoonotic disease without pandemic potential (24), brucellosis can lead to severe complications in humans, including encephalitis, orchitis, arthritis, and myelitis (25). This study provides comprehensive insights into the seroprevalence and risk factors associated with brucellosis in both human and livestock populations in Nyagatare District, Rwanda, using a One Health approach that integrates human, animal, and environmental factors. The findings underscore the endemic nature of brucellosis in this region and highlight critical areas for intervention.

The human seroprevalence observed in this study, using i-ELISA (19.9%, 95% CI: 17.3–22.6), represents the highest ELISA-based prevalence reported to date in Rwanda. This finding is particularly concerning as it suggests a substantial underdiagnosis of brucellosis in human populations in this region, potentially leading to mismanagement of febrile illnesses. This prevalence is comparable to the prevalence previously reported in humans in Uganda (21.2%) (26), likely reflecting the heightened exposure risk among participants, most of whom are part of high-risk populations with regular livestock contact. A recent study conducted in the Kagera region of Tanzania reported a much higher seroprevalence of 41.1% among individuals in a multi-herd ranch system, with the elevated risk attributed to close contact with animals during parturition and handling aborted fetuses (27). This comparison highlights the critical role of occupational exposure and livestock handling practices as significant contributors to human brucellosis risk in endemic regions. Consistently, occupation was a significant risk factor associated with brucellosis seropositivity in our study. Particularly, animal health care practitioners exhibited higher odds of brucellosis infection (OR = 2.90, 95% CI: 1.38–6.06), compared to other occupational groups. Notably, the prevalence of brucellosis observed among veterinary professionals in this study (35.2%) surpassed that reported in India (7.04%) and Turkey (11.8%) in animal health care practitioners (28, 29). This occupation-associated prevalence observed in the current study could be attributed to the heightened risk of exposure inherent to close and regular contact with infected animals. Furthermore, this exposure risk may be exacerbated by a lack of awareness regarding the zoonotic nature of brucellosis among veterinarians and para-veterinarians in Rwanda.

Indeed, although no significant exposure risk factors were identified for veterinarians and butchers, the high seroprevalence observed in these groups suggests inadequate biosafety precautions like slaughtering with bare hands and the occurrence of accidental self-injections during blood sampling and vaccinations against brucellosis (Supplementary Tables S4A, S4B). Similar findings have been reported in studies from Tanzania and Uganda (24–26), emphasizing the need for occupation-specific preventive measures. Thus, it is crucial to continually educate veterinarians, butchers, and their supervisors on biosafety and biosecurity practices when sampling, vaccinating, handling, and treating livestock. The absence of statistically significant risk factors in our study may be attributed to the limited sample size for these two groups (veterinarians and butchers). Therefore, future large-scale investigations focused on these occupational groups are necessary to better understand the specific predictors of brucellosis risk in Rwanda. These findings highlight the urgent need for targeted interventions in high-risk occupational groups and emphasize the importance of biosecurity practices.

In the current study, age and gender were demographic potential risk factors associated with brucellosis in humans. This agreed with findings reported in Uganda by (26). Both studies indicated that males and older individuals demonstrate a stronger association with higher odds of brucellosis seropositivity compared to females and young individuals. In Rwanda, livestock farming remains an important economic activity, with a significant proportion of farms management activities and services being handled by men, compared to women who are predominantly involved in small-scale and/or zero-grazing farming operations (19, 30, 31). This gendered pattern in livestock management and activity distribution could contribute to the observed gender disparity in brucellosis seropositivity, where males seem to be associated with higher odds of infection. These observations demonstrate the importance of considering gender dynamics in understanding and addressing the risk factors associated with brucellosis transmission in both human and livestock populations. The observed AUC values for both the human model (0.76) and livestock model (0.78) further demonstrate good discriminatory performance, supporting the reliability of the identified risk factors. In addition to demographic and occupational factors, questionnaire findings provided important contextual insights. Despite the lack of statistical significance in our dataset, the high prevalence of unpasteurized milk consumption remains a major epidemiological concern, as raw milk is a well-documented transmission route in East Africa. The absence of an observed association here may reflect sampling bias or underreporting, but warrants attention for public health education. Similarly, awareness gaps about brucellosis transmission underscore the need for targeted outreach interventions to improve knowledge and prevention practices in pastoral communities.

This study showed that the *Brucella* seroprevalence increases with age, possibly due to cumulative exposure to risk environments over time. Indeed, older individuals, particularly those working in high-risk occupations such as livestock farming or veterinary services, are more likely to have repeated contact with infected animals or contaminated materials, increasing their odds of seropositivity. The strong age-related gradient observed (OR = 7.39, 95% CI: 3.82–14.8 for those >55 years) highlights the importance of targeting prevention strategies toward long-term livestock workers who may have developed habitual unsafe practices over decades of animal contact. This has been confirmed in studies where middle-aged adults had significantly higher odds of being seropositive compared to younger individuals (32, 33). Similarly, as seen in this and other studies (33, 34) older livestock tend to have a higher prevalence of brucellosis, suggesting that both humans and animals face increased risk due to prolonged exposure.

In livestock, seroprevalence rates of 11.9% (95% CI: 9.4–14.5) in cattle and 11.3% (95% CI: 7.1–15.4) in goats were found, aligning closely with recent reports of 8.2% in cattle (19) and 10.7% in goats (30) within the same district. The similarity in prevalence between cattle and goats is particularly noteworthy as it suggests potential cross-species transmission or common environmental sources of infection, which has important implications for control strategies. However, other studies have reported higher brucellosis seroprevalence rate, such as 22% in cattle and 51.6% in bulk milk from both open grazing and zero-grazing systems (35). These findings collectively suggest that brucellosis is endemic in this transboundary region. The variations in reported prevalence rates across studies may be attributed to differences in sample sizes and other potential factors, such as herd composition and sampling period (23). Additionally, factors such as differences in management practices, geographical variability, and livestock-wildlife interactions could also contribute to these disparities, warranting further investigation into the regional epidemiology of brucellosis.

Our study also provides, the first report of brucellosis seroprevalence in sheep in Rwanda, with a prevalence of 1.4% (95% CI: 0.0–4.2). This finding is comparable to the 1.23% reported in Uganda in 2022 (36). Although the prevalence is low, this finding is significant as sheep may serve as potential reservoirs for *Brucella* infection within mixed herds, especially given that small ruminants are not vaccinated against brucellosis in Rwanda. The significantly lower odds of seropositivity in sheep compared to cattle (OR = 0.12, 95% CI: 0.01–0.60, *p* = 0.042) suggests species-specific differences in susceptibility or exposure that could inform targeted control strategies. However, this prevalence is lower than the average rate of 13.8% reported across the entire East African Community (EAC) (37) and the 12.8% prevalence reported in sheep in South Africa (38). The relatively lower prevalence observed in Rwanda could be attributed to country's small sheep population, reported to be 331,748 (39), as well as the limited sample size in our study. These findings highlight the critical need for enhanced surveillance and control strategies for brucellosis in small ruminants, particularly in Rwanda where these species are not vaccinated. While the lower seroprevalence in sheep (1.4%) compared to goats (11.3%) suggests species-specific transmission dynamics, both species can serve as maintenance hosts in mixed herding systems. The combination of lack of vaccination programs in small ruminants, and the practice of mixed livestock farming in Nyagatare District, create conditions that could facilitate inter-species transmission. Additionally, the identification of shared risk factors such as communal grazing and water points highlights the need for integrated control approaches that consider the interconnected nature of small ruminant management practices in this region.

In the current study, we observed that improper disposal of aborted materials and keeping aborting animals within a herd led to significant increased odds of brucellosis seropositivity (OR = 3.15, 95% CI: 1.18–7.99 and OR = 10.0, 95% CI: 2.27–49.2, respectively) consistent with previous reports (40, 41). Indeed, these inadequate biosecurity practices are well-documented practices that significantly increase the risk of brucellosis transmission (40, 41). This is because *Brucella* bacteria are highly concentrated in aborted tissues, placental fluids, and vaginal discharges of infected animals, making these materials potent sources of infection (40). In our study, we observed that these poor biosecurity practices led to significantly increased odds of brucellosis seropositivity. Similar findings have been reported in other studies, where improper management of aborted materials and the presence of aborting animals in herds were strongly associated with increased seroprevalence of brucellosis. These results highlight the importance of implementing proper disposal methods, such as burying or incinerating aborted materials, and promptly isolating aborting animals to prevent further transmission within herds and to human handlers.

Lastly, there were geographical significant difference in *Brucella* seropositivity in the current study with Rwimiyaga sector exhibiting the highest prevalence (OR = 3.06, 95% CI: 1.37–7.45, *p* = 0.009) compared to the Karangazi sector. Interestingly, *Brucella melitensis* was isolated from a goat flock presenting a storm abortion in this sector in 2018 (42). Thus, this difference may be attributed to the previous exposure and persistence of brucellosis infection in animals of Rwimiyaga sector. The identification of this spatial cluster of brucellosis is a critical finding as it suggests potential environmental or management factors specific to this area that facilitate transmission. This indicates that brucellosis control strategies including vaccination, surveillance and education campaign should prioritize this sector to effectively mitigate brucellosis. Geographic targeting of interventions based on spatial risk assessment can optimize resource utilization in control programs, particularly in resource-limited settings.

## 5 Conclusions

This study demonstrates high seroprevalence of brucellosis among humans (19.9%) and livestock (10.9%) in Nyagatare District. Key risk factors included male gender, older age, and animal health occupations in humans, and poor abortion management practices in livestock. The Rwimiyaga sector emerged as a hotspot of transmission.

Based on these findings, we recommend three specific interventions:

1. Mandatory personal protective equipment and biosafety training for animal health workers.
2. Community education on proper disposal of potentially infectious materials, particularly aborted fetuses.
3. Implementation of sector-specific control strategies targeting Rwimiyaga sector.

These evidence-based recommendations require a coordinated One Health approach involving veterinary, medical, and public health authorities to effectively reduce the burden of brucellosis in this region.

Our findings provide evidence to guide Rwanda's national zoonotic disease control program and emphasize the importance of One Health approaches for brucellosis control in East Africa. Integrating these interventions into Rwanda's National Zoonotic Disease Control Program would strengthen surveillance, prevention, and cross-sectoral coordination to reduce the burden of brucellosis.

## 6 Study limitations

Our study has several limitations that should be considered when interpreting the results. First, the small sample sizes of high-risk occupational groups (veterinarians and butchers) limited our ability to detect occupation-specific risk factors. While this sampling distribution reflects the actual occupational demographics in the district, it reduced statistical power for between-groups comparisons. Although we found elevated seroprevalence among veterinarians (35.2%; Supplementary Table S4), these results should be interpreted conservatively given the wide confidence intervals stemming from the limited sample size.

Second, our human sampling strategy focusing on febrile patients and high-risk occupational groups introduces potential selection bias, and the reported 19.9% seroprevalence likely overestimates the true prevalence in the general population. Third, the cross-sectional design prevents us from establishing temporal relationships between risk factors and infection, and potential seasonal variations in brucellosis transmission could not be assessed. Fourth, while i-ELISA provides high sensitivity and specificity for detecting anti-*Brucella* antibodies, it cannot distinguish between active and past infections, potentially overestimating current disease burden. Finally, we hope that future studies using molecular techniques and longitudinal designs, focusing on veterinary professionals across multiple districts in Rwanda, will better elucidate the epidemiology of brucellosis and help inform targeted interventions.

## Data Availability

The raw data supporting the conclusions of this article will be made available by the authors, without undue reservation.
