# Supplementary material for: Seroprevalence and risk factors associated with brucellosis in humans and livestock in Nyagatare district of Rwanda
Source: Front Public Health. 2025 Sep 26;13:1665341. doi: 10.3389/fpubh.2025.1665341 (PMC12511041; doi:10.3389/fpubh.2025.1665341)
Supplement: Supplementary file 1 [file Data_Sheet_1.pdf]

BruSTAR_Nyagatare

BruSTAR

Form information

Institution

- ○ UR-SPH
- ○ UR-SVM
- ○ NRL

Interviewer ID

Date of interview

Form ID

Location (GPS coordinates)

District

- ○ Nyagatare

Sector

[illegible]

- Karangazi
- Rwempasha
- Rwimiyaga

Cell_Karangazi

- Kamate
- Karama
- Kizirakome
- Mbare
- Musenyi
- Ndama
- Nyagashanga
- Nyamirama
- Rubagabaga
- Rwenyemera
- Rwisirabo

Cell_Rwempasha

- Cyenjonjo
- Gasinga
- Kabare
- Kazaza
- Mishenyi
- Rugarama
- Rukorota
- Rutare

- Rwempasha
- Ryeru

Cell_Rwimiyaga

- Gacundezi
- Kabeza
- Kirebe
- Ntoma
- Nyarupfubire
- Nyendo
- Rutungu
- Rwimiyaga

Village_Kamate

- Bugarama
- Buhongoro
- Kamate
- Kigazi
- Muzehe

Village_Karama

- Karama
- Makomo
- Rundiro

Village_Kizirakome

- Kageyo
- Kahi

- Kizirakome
- Rurebe

Village_Mbare

- Kabirizi
- Kajumo
- Karohoza
- Mbare
- Rwarucura
- Ryabega

Village_Musenyi

- Bwanga
- Gacungiro
- Kabeza
- Musenyi I
- Musenyi II
- Rugarama
- Ruziranyenzi

Village_Ndama

- Akayange
- Ndama
- Rwabiharamba

Village_Nyagashanga

- Bidudu
- Bwera

- Kabare
- Ruhita

Village_Nyamirama

- Kayange I
- Kayange II
- Nkoma I
- Nkoma II
- Nyamirama I
- Nyamirama II

Village_Rubagabaga

- Iraba
- Nkuna
- Nyarutovu
- Rebero

Village_Rwenyemera

- Bwera
- Imishongi
- Kayishunika
- Kizirakome
- Rwenyana
- Rwenyemera
- Rwimirama

Village_Rwisirabo

- Gakoma

- Humure
- Karangazi
- Rubona
- Rukundo

Village_Cyenjonjo

- Cyenjonjo I
- Cyenjonjo II
- Rutare II

Village_Gasinga

- Gasinga
- Nyendo
- Rwibishorogoto

Village_Kabare

- Gituro
- Kabare
- Ururimbi

Village_Kazaza

- Gakindo
- Kazaza
- Rukiri

Village_Mishenyi

- Gicwamba
- Kinungu
- Mishenyi

Village_Rugarama

- Bubare
- Rugarama

Village_Rukorota

- Bukonji
- Rukorota

Village_Rutare

- Mashaka
- Nshuli
- Rutare

Village_Rwempasha

- Nyarubare
- Rwahi
- Rwempasha
- Uwinkiko

Village_Ryeru

- Bweya
- Ryeru

Village_Gacundezi

- Bugaragara
- Gacundezi I
- Gacundezi II
- Rukundo I
- Rukundo II

- ○ Rukundo III

Village_Kabeza

- ○ Gatovu
- ○ Kabeza
- ○ Kabeza Centre
- ○ Kavumu
- ○ Rugarama
- ○ Rukiri I
- ○ Rukiri II

Village_Kirebe

- ○ Gatebe I
- ○ Gatebe II
- ○ Kirebe
- ○ Rukindo

Village_Ntoma

- ○ Gashwenu
- ○ Kibuye
- ○ Kimaramu
- ○ Nyampire
- ○ Rwembogo

Village_Nyarupfubire

- ○ Kamagiri
- ○ Nyakagando I
- ○ Nyakagando II

- Nyarupfubire I
- Nyarupfubire II
- Rwimiyaga I
- Rwimiyaga II

Village_Nyendo

- Isangano
- Nyamirama
- Rebero
- Remera

Village_Rutungu

- Bwera
- Cyamunyana
- Gakagati I
- Gakagati II
- Rubira

Village_Rwimiyaga

- Byimana
- Gakoma
- Kizungu
- Mahoro
- Muyange
- Rebero
- Rwinyange

Gender

- Male
- Female

Age category

- 18-25
- 26-35
- 36-45
- 46-55
- Over 55

Marital status

- Married
- Single
- Divorced/Separated
- Widow
- Not applicable

Role in the household

- Parent
- Child
- Employee (homemaker)
- Family relative

Education

- Never went to school
- Primary school
- Middle school
- High school

- ○ College (University)

Occupation

- ☐ Farmer
- ☐ Farm assistant
- ☐ Human health care practitioner
- ☐ Animal health care practitioner
- ☐ Student
- ☐ Teacher
- ☐ Officer worker
- ☐ Retired
- ☐ Other

Other occupation

How many people live in your household?

In the past 30 days, have you slept in the same room or enclosure as any of the following animals ?

- ☐ Cattle
- ☐ Goats
- ☐ Sheep
- ☐ None of the above

In the past 30 days, have you participated in any of the following activities?

- ☐ Milked livestock

- [ ] Herded livestock
- [ ] Came in contact with livestock waste
- [ ] Assisted in livestock parturition
- [ ] Handled aborted livestock fetus
- [ ] Slaughtered or butchered livestock
- [ ] None of the above

In the past 30 days, which of the following meats or offal have you consumed?

- [ ] Raw meat or offal from cattle
- [ ] Raw meat or offal from goat
- [ ] Raw meat or offal from sheep
- [ ] Raw meat or offal from another animal:
- [ ] None of the above

Other animal for which meat or offal was consumed

In the past 30 days, which of the following blood meals have you consumed?

- [ ] Raw cattle blood
- [ ] Raw goat blood
- [ ] Raw sheep blood
- [ ] Raw blood from another animal
- [ ] None of the above

Other animals for which blood meals were consumed

In the past 30 days, which of the following dairy products have you consumed?

- [ ] Pasteurized or boiled milk
- [ ] Raw milk
- [ ] Cheese made from raw milk
- [ ] Butter or cream made from raw milk
- [ ] Yogurt made from raw milk
- [ ] Other food prepared by adding raw milk before cooking
- [ ] None of the above

Other animals for which blood meals were consumed

If any raw milk or any products from raw milk were consumed, where did those come from?

- [ ] Shop
- [ ] Open market
- [ ] Neighbors/friends/family
- [ ] From own cow
- [ ] From own goats
- [ ] From own sheep

In the past two weeks, have you or any of your family members had any of the following symptoms?

- [ ] Fever
- [ ] Night sweats
- [ ] Fatigue

- ☐ Joint pain
- ☐ Swollen joints
- ☐ Muscular pain
- ☐ Back pain
- ☐ Headaches
- ☐ Loss of appetite
- ☐ None of the above

If you or your family member had fever:

When was this (fever)?

- ○ In the past two weeks
- ○ In the past month
- ○ A few months ago

Type of fever

- ○ Continuous
- ○ Intermittent

Fever duration (in days)

If you had fever that lasted for more than 3 days, did you seek professional healthcare?

- ☐ Yes
- ☐ No

Were you hospitalized?

- Yes
- No

Have you recovered from the illness?

- Yes
- No
- Unsure

Is the respondent a female?

- Yes
- No

Have you ever been pregnant?

- Yes
- No

How many live births have you had?

Have you ever had a miscarriage or stillbirth?

- Yes
- No

Which of the following professions apply to the respondent?

- Livestock keeper (farm owner or works on farm)
- Butcher or works at a slaughterhouse
- Veterinarian / para-veterinarian
- None of the above

Number of cattle on farm

Males

Females

Lactating females

Number of sheep on farm

Males

Females

Lactating females

Number of goats on farm

Males

Females

Lactating females

Production mode

- ○ Pastoralism (mobility, exclusively cattle, no crops farming)
- ○ Agro-Pastoralism (both crops and livestock)
- ○ Commercial (Ranchers, Dairy farms, fenced farms)
- ○ Smallholder dairy systems (principal output is “milk for sale”)

Do animals live in the same house as people in the household?

- ○ Yes
- ○ No

Is the farm fenced?

- ○ Yes
- ○ No

Do you or your animals interact with wildlife animals in any way?

- ○ Yes
- ○ No

Do you take your animals to drink water at water points shared with other farmers in the area?

- Yes
- No

Do you fetch animals' drinking water from wanter points shared with other farmers in the area?

- Yes
- No

What breeding methods are used?

- Natural (using bulls)
- Artificial insemination
- Both methods are used

Where are the used bulls from?

- Own bulls on the same farm
- Bull sharing with neighbors (borrowing and/or renting bulls)
- Other

Other sources of bulls

Do you have a veterinarian who takes care of your animals?

- Yes
- No

Who takes care of the animal health on your farm?

- We do it ourselves
- Other farmers who are more knowledgeable in animal health

○ Other

Other animal health care provider

Have you ever had infertility issues in your herd?

○ Yes

○ No

When there is a case of abortion, how do you dispose of aborted materials?

☐ Not applicable (no abortions observed)

☐ Burying them in the ground

☐ Feeding them to other animals like dogs

☐ Throwing them to the field without burying them

☐ Other

Other disposal means

What happens to the animal that has a case of abortion?

○ Not applicable (no abortions observed)

○ Sold for slaughter

○ Sold to other famers

○ Kept in the herd

Have recently introduced new animal/animals in your herd?

○ Yes

○ No

When did you introduce new animal(s). Choose all that apply if multiple introductions.

- ☐ 0 - 3 months ago
- ☐ 4 - 6 months ago
- ☐ 7 months - 2 years ago
- ☐ More than 2 years ago

How did you procure the new animal(s)?

- ☐ From neighbor farmers
- ☐ From local markets
- ☐ From other countries
- ☐ Dowry
- ☐ Other:

Other sources of animals

Do you get the animal tested for diseases before it is introduced into your herd?

- ○ Yes
- ○ No

Are you aware of a disease called brucellosis?

- ○ Yes
- ○ No

Were your animals vaccinated against brucellosis in last two years?

- ○ Yes
- ○ No

Do you have your animals regularly tested for brucellosis?

- Yes
- No

Are you aware of a disease called brucellosis that can affect animals and humans?

- Yes
- No

Are you aware that brucellosis can be transmitted through animal products like meat and milk?

- Yes
- No

Do you ever eat the meat that has been condemned at the slaughterhouse?

- Yes
- No

Have you ever had cuts on you hands while handling the meat?

- Yes
- No

How often does this happen (getting cuts on hands)

- Very often
- It happened only once or twice

Do you keep working in meat handling when you have cuts on your hands?

- Yes
- No

Do yo always wear a face mask when handling meat?

- Yes
- No

Do you always wear gloves when handling meat?

- Yes
- No

Does it happen for you to eat while working at the slaughterhouse or butchery?

- Yes
- No

Do you always wear PPE like face masks, gloves and eye goggles when assisting in normal animal calving?

- Yes
- No
- Not applicable (I do not assist in normal calving)

Do you ever handle dystocia cases or do caesareans without recommended PPE (face mask, goggles, gloves)?

- Yes
- No
- Does not apply (not involved in veterinary obstetrics)

Have ever had an accidental self-injection with a needle while vaccinating animals

- Yes
- No

Have you ever had an accidental self-injection with a needle while collecting blood from animals?

- Yes
- No
