# Supplementary material for: Seroprevalence and risk factors associated with brucellosis in humans and livestock in Nyagatare district of Rwanda
Source: Front Public Health. 2025 Sep 26;13:1665341. doi: 10.3389/fpubh.2025.1665341 (PMC12511041; doi:10.3389/fpubh.2025.1665341)
Supplement: Supplementary file 2 [file Data_Sheet_2.pdf]

S2 Table. Univariable logistic analysis of the risk factors for brucellosis in whole human participants (N=886)

Variables	Categories	Interviewed	i-ELISA (%)	P value
Sector	Rwempasha	315	58 (18.4)	0.626
	Rwimiyaga	344	71 (20.6)	
	Karangazi	227	49 (21.6)	
Gender	Female	221	22 (9.9)	0.000
	Male	665	156 (23.5)	
Age	18-25	187	19 (10.2)	0.000
	26-35	276	39 (14.1)	
	36-45	186	40 (21.5)	
	46-55	137	42 (30.7)	
	≥55	100	38 (38.0)	
Marital status	Married	587	137 (23.3)	0.007
	Single	275	39 (14.2)	
	Divorced	3	0 (0.0)	
	Widow	21	2 (9.5)	
Education	No education	173	47 (27.2)	0.008
	Primary	417	76 (18.2)	
	Middle	100	13 (13.0)	
	High school	124	21 (16.9)	

	College	72	21 (29.2)	
Occupation	Animal Healthcare practitioner	54	19 (35.2)	0.000
	Human Healthcare practitioner	36	7 (19.4)	
	Livestock farmers	442	104 (23.4)	
	Meat handlers	153	15 (9.8)	
	Milk handlers	186	30 (16.1)	
	Others	15	3 (20.0)	
Sleeping with animals	Cattle	4	0 (0.0)	0.430
	Goats	3	1 (33.3)	
	None of the above	879	177 (20.1)	
Milking livestock	Yes	412	113 (27.4)	0.000
	No	474	65 (13.7)	
Herding livestock	Yes	238	73 (30.7)	0.000
	No	648	105 (16.2)	
Contact with waste	Yes	302	91 (30.1)	0.000
	No	584	87 (14.9)	
Assisted parturition	Yes	149	46 (30.9)	0.001
	No	737	132 (17.9)	
Handled aborted fetus	Yes	157	61 (38.9)	0.000
	No	729	117 (16.1)	
Slaughtered livestock	Yes	168	21 (12.5)	0.005

	No	718	157 (20.1)	
Consumption of pasteurized/boiled milk	Yes	436	86 (19.7)	0.800
	No	450	92 (20.4)	
Consumption of raw milk	Yes	441	94 (21.3)	0.410
	No	445	84 (18.9)	
Consumption of cheese from raw milk	Yes	6	0 (0.0)	0.600
	No	880	178 (20.2)	
Consumption of butter from raw milk	Yes	6	0 (0.0)	0.600
	No	880	178 (20.3)	
Symptom of fever	Yes	111	12 (10.8)	0.013
	No	775	166 (21.4)	
Symptom of night sweats	Yes	62	10 (16.1)	0.520
	No	824	168 (20.4)	
Symptom of fatigue	Yes	136	19 (13.9)	0.062
	No	750	159 (21.2)	
Symptom of joint pain	Yes	125	34 (27.2)	0.040
	No	761	144 (18.9)	

Symptom of swollen joints	Yes	66	18 (27.3)	0.150
	No	820	160 (19.5)	
Symptom of muscular pain	Yes	89	22 (24.7)	0.260
	No	797	156 (19.6)	
Symptom of back pain	Yes	203	40 (19.7)	0.920
	No	683	138 (20.2)	
Symptom of headaches	Yes	251	40 (15.9)	0.065
	No	635	138 (21.7)	
Symptom of loss of appetite	Yes	75	11 (14.7)	0.290
	No	811	167 (20.6)	
When fever occurred	In the past two weeks	107	11 (10.3)	0.020
	In the past month	5	1 (20.0)	
	A few months ago	1	0 (0.0)	
	NA	773	166 (21.5)	
Seeking Health care	Yes	77	6 (7.8)	0.010
	No	17	3 (17.7)	
	NA	792	169 (21.4)	
Type of fever	Continuous	18	0 (0.0)	0.002

	Intermittent	89	9 (10.1)	
	NA	779	169 (21.7)	
Hospitalization	Yes	5	0 (0.0)	0.800
	No	878	178 (20.3)	
	NA	3	0 (0.0)	
Recovered	Yes	4	0 (0.0)	0.600
	No	1	0 (0.0)	
	NA	881	178 (20.2)	
Pregnant	Yes	169	18 (10.7)	0.000
	No	37	2 (5.4)	
	NA	680	158 (23.2)	
Miscarriage or stillbirth	Yes	43	4 (9.3)	0.004
	No	127	15 (11.8)	
	NA	716	159 (22.2)	
Sampling area	Health center	196	22 (11.2)	0.001
	Other sampling areas	690	156 (22.6)	
Awareness of brucellosis	Yes	282	81 (28.7)	0.000
	No	134	19 (14.2)	
	NA	470	78 (16.6)	
Awareness of zoonotic brucellosis	Yes	36	1 (2.8)	0.000

	No	132	15 (11.4)
	NA	718	162 (22.6)
