# Supplementary material for: Seroprevalence and risk factors associated with brucellosis in humans and livestock in Nyagatare district of Rwanda
Source: Front Public Health. 2025 Sep 26;13:1665341. doi: 10.3389/fpubh.2025.1665341 (PMC12511041; doi:10.3389/fpubh.2025.1665341)
Supplement: Supplementary file 3 [file Data_Sheet_3.pdf]

S3 Table. Univariable regression analysis of the potential risk factors for livestock brucellosis in Nyagatare district, Rwanda

Variables	Categories	Interviewed	i-ELISA	p-value
Age	Adult (≥5 years)	861	100 (11.61%)	0.08
	Young adult (3-4 years)	26	1 (3.85%)	
	Young (1-2 years)	43	1 (2.33%)	
Sex	Female	844	98 (11.61%)	0.05
	Male	86	4 (4.65%)	
Sector	Karangazi	343	30 (8.75%)	0.00001
	Rwempasha	288	16 (5.56%)	
	Rwimiyaga	299	56 (18.97%)	
Breed	Ankole	4	0 (0.0%)	0.04
	Cow cross	597	74 (12.4%)	
	Cow exotic	36	1 (2.78%)	
	Exotic breed goat	13	3 (23.08%)	
	goat cross	18	1 (5.56%)	

	goat local	193	22 (11.4%)	
	sheep cross	11	0 (0.0%)	
	sheep local	58	1 (1.72%)	
Education	College	48	8 (16.67%)	0.007
	High school	215	16 (7.44%)	
	Middle school	80	3 (3.75%)	
	Never went to school	278	42 (15.11%)	
	Primary school	309	33 (10.68%)	
Occupation	Farm Assistant	445	40 (8.99%)	0.2
	Farmer	421	56 (13.30%)	
	Human health care practitioner	9	0 (0.0%)	
	Officer worker	55	6 (10.91%)	
Health status	Aborted	82	22 (26.83%)	0.004
	aborted and repeat breeder	17	3 (17.65%)	
	Blind	4	0 (0.0%)	

	Emaciated	1	0 (0.0%)	
	Hygroma	1	0 (0.0%)	
	Orf	1	0 (0.0%)	
	Repeat breeder	82	22 (26.83%)	
	Retained placenta	2	0 (0.0%)	
	Stillbirth	1	0 (0.0%)	
	Healthy	720	69 (9.58%)	
Lactating	Aborted	1	0 (0.0%)	0.9
	Yes	481	31 (6.44%)	
	No	447	27 (6.04%)	
Animals living with people	Yes	12	0 (0.0%)	
	No	918	102 (11.11%)	0.4
Fenced farm	Yes	845	98 (11.60%)	0.12
	No	73	4 (5.48%)	
	Yes	765	92 (12.03%)	0.03

Infertility history	No	165	10 (6.06%)	
Aborted animal fate	Kept in the herd	807	579 (71.75%)	0.00001
	Sold for slaughter	40	40 (100%)	
	NA (no abortion cases)	83	71 (85.54%)	
Animal feed abort	Yes	556	75 (13.49%)	0.003
	No	374	27 (7.22%)	
Burying aborted	Yes	288	15 (5.21%)	0.0003
	No	642	87 (13.55%)	
Throwing abort	Yes	166	33 (19.88%)	0.0002
	No	764	69 (9.03%)	
Breeding methods	Artificial insemination	53	1 (1.89%)	0.03
	Using Bull	850	100 (11.76%)	
	Both methods	27	1 (3.70%)	
Origin of the bull	Sharing with neighbors	39	0 (0.0%)	0.02
	Own bull at the farm	838	101 (12.05%)	

Shared water	Yes	314	33 (10.51%)	0.8
	No	616	69 (11.20%)	
Fetch water at water points	Yes	33	3 (9.09%)	1
	No	583	66 (11.32%)	
Having a veterinarian	Yes	268	31 (11.57%)	0.8
	No	635	69 (10.87%)	
New introduction	Yes	439	37 (8.43%)	0.03
	No	491	65 (13.24%)	
Introduction origin	Dowry	54	5 (7.41%)	0.0034
	From local markets	86	1 (1.16%)	
	From neighbor farmers	174	15 (8.62%)	
	Other country	17	0 (0.0%)	
	Other districts	108	17 (15.74%)	
Introduction time	0 - 3 months ago	347	26 (7.49%)	
	4 - 6 months ago	92	11 (11.96%)	0.2

Production mode	Agro-pastoralism	34	1 (2.94%)	0.2
	Commercial (dairy farms)	856	92 (10.75%)	
Vaccination	Yes	126	14 (11.11%)	0.02
	No	697	84 (12.05%)	
	Unknown	107	4 (3.74%)	
Wildlife interaction	Yes	346	41 (11.85%)	0.5
	No	584	61 (10.45%)	
Brucellosis awareness	Yes	823	98 (11.91%)	0.008
	No	107	4 (3.74%)	
