# Supplementary material for: Seroprevalence and risk factors associated with brucellosis in humans and livestock in Nyagatare district of Rwanda
Source: Front Public Health. 2025 Sep 26;13:1665341. doi: 10.3389/fpubh.2025.1665341 (PMC12511041; doi:10.3389/fpubh.2025.1665341)
Supplement: Supplementary file 4 [file Data_Sheet_4.pdf]

S4A Table. Univariable logistic analysis of the risk factors for brucellosis in veterinarians

Variables	Categories	Interviewed	i-ELISA (%)	P value
Wearing PPEs when calving	Yes	21	10 (47.6)	0.900
	No	19	8 (42.1)	
Wearing PPES during surgery	Yes	26	11 (42.3)	0.700
	No	14	7 (50.0)	
Self-injection during sampling	Yes	30	15 (50.0)	0.460
	No	10	3 (30.0)	
Self–injection during vaccination	Yes	32	15 (46.9)	0.700
	No	8	3 (37.5)	

S4B. Table. Univariable logistic analysis of the risk factors for brucellosis in butchers

Variables	Categories	Interviewed (%)	i-ELISA (%)	P value
Hand cuts	Yes	137 (81.55)	12 (8.8)	0.500
	No	31 (18.45)	4 (12.9)	
Frequency of hand cuts	Once or twice	41 (24.4)	4 (9.8)	0.600
	Very often	96 (57.1)	8 (8.3)	
	NA (no hand cuts)	31 (18.5)	4 (12.9)	
Working with hand cuts	Yes	112 (66.7)	9 (8.0)	0.600
	No	25 (14.9)	3 (12.0)	
	NA (no hand cuts)	31 (18.5)	4 (12.9)	
Eating at work	Yes	125 (74.4)	11 (8.8)	0.500
	No	43 (25.6)	3 (11.6)	

Wearing gloves	Yes	4 (2.4)	0 (0.0)	1.000
	No	164 (97.6)	16 (9.8)	
Wearing facemask	Yes	4 (2.4)	0 (0.0)	1.000
	No	163 (97.0)	16 (9.8)	
	NA (no answer)	1 (0.6)	0 (0.0)	
Awareness of brucellosis transmission	Yes	8 (4.8)	1 (12.5)	1.000
	No	28 (16.7)	0 (0.0)	
	NA (no answer)	132 (78.6)	15 (11.4)	
Zoonotic brucellosis Awareness	Yes	36 (21.4)	1 (8.8)	0.200
	No	132 (78.6)	15 (11.6)	
